# Measurement of Cognition for the National Children's Study

**DOI:** 10.3389/fped.2021.603126

**Published:** 2021-05-31

**Authors:** Philip David Zelazo, Stella F. Lourenco, Michael C. Frank, Jed T. Elison, Robert K. Heaton, Henry M. Wellman, Jerry Slotkin, Maria Kharitonova, J. Steven Reznick

**Affiliations:** ^1^Institute of Child Development, University of Minnesota, Minneapolis, MN, United States; ^2^Department of Psychology, Emory University, Atlanta, GA, United States; ^3^Department of Psychology, Stanford University, Palo Alto, CA, United States; ^4^Department Psychiatry, University of California, San Diego, San Diego, CA, United States; ^5^Department of Psychology, University of Michigan, Ann Arbor, MI, United States; ^6^Center for Health Research and Translation, University of Delaware, Newark, DE, United States; ^7^xSEL Labs, Evanston, IL, United States; ^8^Department of Psychology, University of North Carolina at Chapel Hill, Chapel Hill, NC, United States

**Keywords:** cognition, measurement, longitudinal, environment, tablet-based

## Abstract

The National Children's Study Cognitive Health Domain Team developed detailed plans for assessing cognition longitudinally from infancy to early adulthood. These plans identify high-priority aspects of cognition that can be measured efficiently and effectively, and we believe they can serve as a model for future large-scale longitudinal research. For infancy and toddlerhood, we proposed several paradigms that collectively allowed us to assess six broad cognitive constructs: (1) executive function skills, (2) episodic memory, (3) language, (4) processing speed, (5) spatial and numerical processing, and (6) social cognition. In some cases, different trial sequences within a paradigm allow for the simultaneous assessment of multiple cognitive skills (e.g., executive function skills and processing speed). We define each construct, summarize its significance for understanding developmental outcomes, discuss the feasibility of its assessment throughout development, and present our plan for measuring specific skills at different ages. Given the need for well-validated, direct behavioral measures of cognition that can be used in large-scale longitudinal studies, especially from birth to age 3 years, we also initiated three projects focused on the development of new measures.

## Introduction

The United States National Children's Study (NCS), authorized by the Children's Health Act of 2000, was designed to examine environmental influences on health and development. From 2009 to 2014, a pilot study (the NCS Vanguard Study) for a planned large-scale longitudinal cohort investigation enrolled over 14,000 participants in 5,000+ families and collected more than 14 million records, including biological and environmental samples. These data are available to qualified researchers via the NCS Archive (NOT-HD-16-005), now located in NICHD's Data and Specimen Hub (DASH). The NCS was terminated by the NIH Director in December 2014, due to concerns about the magnitude, complexity, and long-term feasibility of the originally envisioned project, but prior to this, plans were made to conduct the larger, main study, in which 100,000 children would be assessed longitudinally from before birth to age 21 years. These plans included assessment of a wide range of environmental, biological, and behavioral variables, spanning broad domains of health and human functioning. The current article focuses on the rationale and plans for assessing cognition throughout development. These plans identify high-priority aspects of cognition that can be measured efficiently and effectively at a wide range of ages, and provide a useful model for future large-scale longitudinal research on the development of cognition across the lifespan. The use of comparable measures across studies, and in different countries, has the potential to add greatly to a richer and more globally representative characterization of cognitive development in relation to both environmental and biological influences, and to important functional outcomes.

Cognition, or thinking, spans a range of information processing skills involved in learning, memory, communication, and problem solving. These skills are essential for healthy adaptation in society, and individual differences in these skills predict important developmental outcomes, including educational achievement, innovation and job success, and parenting and interpersonal relationships (1). Impairments in cognitive skills are markers of numerous disorders emerging in childhood, adolescence, and adulthood (2).

As part of the NCS, the authors of this article were convened to comprise the NCS Cognitive Health Domain Team. Collectively, this team has considerable expertise in the scientific study of a wide range of cognitive skills across the lifespan. The team was charged with proposing and designing assessments of neurocognitive development (from birth to adulthood) that have potential to inform high-priority policy goals, such as the early identification and amelioration of neurodevelopmental disorders, and the amelioration of systemic inequities in developmental outcomes. The proximal aim was to examine influences on key aspects of cognition and the dynamic ways in which cognitive development interacts with processes at other levels of analysis (e.g., environment, brain, genes, epigenetic processes).

Our team proposed to assess six broad cognitive constructs, or types of cognitive skill. These constructs were selected based on an extensive review of research with infants and young children that has shown both that these constructs can be measured directly (behaviorally) in infancy and that they predict important functional outcomes, including educational achievement, and physical and mental health. Assuming 15 min total for the assessment of cognition at each in-person visit and at each age tested, and that not all constructs would be assessed at each visit, we then designed a paradigm-based approach for infants and toddlers, in which participants are assessed using a series of brief (2–3 min) testing paradigms (e.g., visual search for virtual objects that disappear behind a virtual screen; a looking-while-listening assessment of word comprehension). In some cases, several different skills (e.g., cognitive flexibility, inhibitory control, and processing speed) can be assessed simultaneously. That is, different aspects of children's behavioral responses in a single situation can yield measures of different cognitive constructs and skills within those constructs, allowing for increased efficiency in the collection of relevant data.

Paradigms were designed to be presented on a computer tablet, and we planned to record participants' eye movements in response to events during assessments of specific skills. Looking-time measures are especially useful in early infancy, when infants cannot respond verbally and their range of motor responses is limited. For example, at 8 months of age, we planned to employ a computer tablet-based visual delayed-response paradigm, in which objects are (virtually and conspicuously) hidden at one of the locations and then, after a delay, infants are allowed to search for them by looking to a correct location (or at 21 months, by reaching). We planned to vary the duration of the delay and the number of hiding locations to assess how much information can be kept in mind for how long. Performance on switch trials (i.e., when the location of the hidden object is changed after a series of trials at the same location) provides a measure of cognitive flexibility and inhibitory control. Across trials, infants' reaction times to fixate the correct location provide an index of processing speed.

We proposed to assess the following broad cognitive constructs in this paradigm-based way: (1) executive function skills, (2) episodic memory (learning and recall), (3) language, (4) processing speed, (5) spatial and numerical processing, and (6) social cognition. These six types of cognitive skill can be measured straightforwardly starting in infancy, and early individual differences in these skills predict important developmental outcomes, including educational achievement and social functioning. For example, executive function skills measured in childhood have been found to help predict physical health, substance dependence, socioeconomic status, and the likelihood of a criminal conviction at age 32 years, even after controlling for social class of origin and intelligence (IQ) (3). Childhood impairments in executive function skills are markers of numerous disorders emerging in childhood and adolescence. For example, impairments in executive function skills are associated with Attention Deficit/Hyperactivity Disorder, autism spectrum disorder, and Conduct Disorder, among many other conditions (4–6).

In what follows, we define each type of cognitive skill, summarize its significance for understanding functional outcomes across development, discuss the feasibility of its assessment at different ages, and present our plan for measuring specific skills longitudinally from early infancy into adulthood in the context of the planned NCS. We aimed to assess development as continuously as possible from late infancy into adulthood, but specific ages were selected for assessment based on previous research in cognitive development and the need to coordinate our plans with other plans designed for the NCS as a whole

For executive function skills, episodic memory, language, and processing speed, our paradigm-based approach to assessment in infancy and early childhood can be complemented by, and then replaced by, the NIH Toolbox® Cognition Battery, which is presented on a computer tablet (7), and is available here: https://www.healthmeasures.net/explore-measurement-systems/nih-toolbox/intro-to-nih-toolbox/cognition. We also describe three ongoing projects, launched as part of the NCS, that are designed to create new, well-validated, direct behavioral measures of cognition that can be used in future large-scale longitudinal studies, especially from birth to age 3 years. Further information about specific measures can be obtained from the authors, and by consulting the deliverables from the team's planning process (e.g., more detailed plans and examples), which are in the public domain and can be found at: https://www.nichd.nih.gov/research/supported/NCS.

## Executive Function Skills

### Definition

Executive function skills are the set of attention-regulation skills involved in the deliberate, goal-directed self-regulation of attention and behavior (8). These skills, which depend on the integrity of neural networks involving prefrontal cortex, the anterior cingulate cortex, and other regions [e.g., (9)], include *cognitive flexibility* (i.e., shifting attention flexibly to consider multiple perspectives or to switch between strategies), *inhibitory control* (i.e., deliberately suppressing attention to distractors and refraining from pre-potent impulsive or automatic responding), and *working memory* (i.e., maintaining attention to information in mind and manipulating it during a limited time delay) (10). Executive function skills emerge during the first year of life, develop rapidly between about 2 and 6 years of age, and during the transition to adolescence, and continue to develop into adulthood (11). When considered across the lifespan, executive function skills follow an inverted-U-shaped curve, rising, reaching a peak during the early- or mid-20s, and then falling (12). Executive function deficits in mid- to late- adulthood, such as unwanted intrusions of irrelevant material into one's speech, are signs of neurocognitive decline [e.g., (8, 13)].

### Significance

Executive function skills are required for learning and adjustment to school, where children must attend selectively and ignore distractions, keep information in mind, follow rules, suppress impulses to play or aggress, and use imagination creatively and flexibly to solve problems (14). As noted in the Introduction, impairments in executive function skills are markers of numerous disorders emerging in childhood and adolescence (4–6), and individual differences in children's executive function skills are concurrently and predictively related to academic achievement, including math and reading skills [e.g., (15–17)], and to other important functional outcomes including physical health (3). Executive function skills are also strongly positively related to socioeconomic status in childhood [e.g., (18, 19)]. Further research is required to understand the nature of these associations, but there is growing evidence that prefrontally mediated skills, which modulate many other neural functions, may be especially vulnerable to disruption given their dependence on the integrity of these other neural functions, their protracted developmental course, and their sensitivity to the effects of stress [e.g., (4, 6, 20, 21)]. At the same time, however, in children who are at risk for maladaptation, including for low academic achievement and school drop-out, better executive function skills are associated with better outcomes, suggesting that executive function skills are a protective factor and a sign of resilience in the face of adversity [e.g., (22)]. Moreover, the protracted development of executive function skills suggests a relatively long period of neuroplasticity, and evidence indicates that executive function skills can be improved through training in childhood and beyond (23). Executive function skills are therefore seen as potentially critical to addressing socioeconomic, racial, and other “gaps” in opportunity, achievement, and health outcomes, and they are an increasingly popular target of prevention and early intervention programs.

### Feasibility

Inhibitory control is essential for *sustained selective attention* (24), and both cognitive flexibility and inhibitory control are needed for *future-oriented planning*, including the predictive *anticipation* of future events (25). These specific skills can be measured straightforwardly in infancy using a simple looking-time procedure presented on a computer tablet (see section Project 1: Developmental Cognitive Profiler (DCP): Design and Creation of a Computer Tablet-Based Assessment of Cognitive Constructs in Infants and Toddlers). For example, it is possible to use eye tracking to measure sustained visual attention to sequences of events that end with a rewarding display, as well as anticipatory looking ahead to future locations when a spatial pattern becomes predictable. At 14 and 24 months, working memory, cognitive flexibility, and inhibitory control can be measured using a tablet-based visual or manual delayed response paradigm [e.g., (26)], which also provides measures of sustained attention and processing speed. For older children, the NIH Toolbox Cognition Battery includes brief (<5 min each) tablet-based measures of working memory (List Sorting Working Memory Test), cognitive flexibility (Dimensional Change Card Sort; DCCS), and inhibitory control (Flanker Inhibitory Control and Attention Test; Flanker) that are available for research purposes, validated, normed, available in Spanish, English, and other languages, and suitable for use across the lifespan, starting as young as 3 or 4 years (27, 28). Downward extensions (DEXTs) of these measures, designed to be continuous with the NIH Toolbox measures, make these tasks more suitable for younger children [(29); see section Project 3: Executive Function Formative Project: Design and Creation of a Developmental Extension (DEXT) of the Executive Function (EF) Measures of the NIH Toolbox Cognition Battery].

### Executive Function Measurement Plan

Table 1 shows the measurement plan for this construct. We planned to measure sustained selective attention, cognitive flexibility, and inhibitory control at 8, 14, and 21 months, and predictive (or proactive) attention (i.e., anticipation) at 8 and 14 months, using two paradigms (delayed response and the presentation of predictable patterns, respectively). The NIH Toolbox DCCS would be used to measure cognitive flexibility administered at 3, 4, 5, and 7 years, and then again at 11, 15, and 19 years. The NIH Toolbox Flanker would be used to measure inhibitory control administered at 3, 4, 5, and 9 years, and then again at 13, 17, and 21 years. We planned to measure working memory at 8, 14, and 21 months (using delayed response), and then again at 3, 4, 5, 9, 13, 17, and 21 years using the NIH Toolbox List Sorting Working Memory Test.

**Table 1:** National Children's Study measurement plan for executive function skills.

**Domain**	**Construct**	**Measure**	**0–1 month**	**8 months**	**14 months**	**21 months**	**3 years**	**4 years**	**5 years**	**7 years**	**9 years**	**11 years**	**13 years**	**15 years**	**17 years**	**19 years**	**21 years**
Cognition	Executive function	Sustained attention		X	X	X											
Cognition	Executive function	Proactive/Predictive attention		X	X												
Cognition	Executive function	Cognitive flexibility		X	X	X	X	X	X	X		X		X		X	
Cognition	Executive function	Inhibitory control		X	X	X	X	X	X		X		X		X		X

## Episodic Memory

### Definition

*Episodic memory* refers to the conscious recollection of past experiences, and is needed for the development of a coherent sense of self, as well as many other important functions (30). This form of memory is also important for learning and future planning. Episodic memory involves encoding, storing, and retrieving information. These processes are interconnected, but they can be described as clusters based on various parameters of memory functionality. From a developmental perspective, one of the earliest emerging clusters is *recognition memory*, defined as differentiating between previously experienced information and novel information. Recognition memory, which provides a measure of encoding and storage in the absence of retrieval demands, continues to develop during infancy and toddlerhood, and appears necessary for the healthy, long-term development of numerous complex cognitive and social skills [e.g., (31)]. Early recognition memory is typically measured using procedures that estimate recognition memory capacity based on the type and amount of information that can be represented, the amount of familiarization needed to form a representation, and the duration of storage. In older children and adults, episodic memory is typically assessed using cued recall or free recall paradigms in addition recognition memory, to distinguish among different processes involved (e.g., storage vs. retrieval). Participants might be asked to reproduce previously experienced events through verbal report or imitative action, for example.

### Significance

As noted, episodic memory is essential for a range of functions, and impairments in episodic memory, as seen in cases of anterograde amnesia, are devastating for the individuals involved (32). Individual differences in episodic memory in infancy predict key cognitive outcomes later in life. For example, recognition memory for a series of objects after delays of 1, 3, or 5 min at age 7 months demonstrates the encoding and storage of information, and has been linked to executive function skills assessed at 11 years (33). Recognition memory is affected by brain insults, such as febrile seizures (34), and developmental impairments in recognition memory have important consequences for children's lives. Examples include studies linking poor recognition memory at 7–9 months with perceptual performance at 4.5 years (35), and recognition memory at 6–12 months with adult IQ and academic achievement (31).

### Feasibility

Recognition memory can be measured straightforwardly in infancy, during the toddler years, and across development into adulthood. The standard procedure for assessing recognition memory in infants is relatively simple, and is based on research conducted in the early 1970s: two identical visual stimuli are presented briefly then removed. After a brief delay, one of the original stimuli reappears paired with a novel stimulus, and recognition memory is assessed based on visual preference (looking time) for the novel stimulus [(1), for review].

Recognition memory capacity is defined based on parameters, such as the type of information, the duration of initial access to the information, or the duration of storage of the information. Recognition memory capacity typically reaches extremely high levels by 2 years, so we proposed only to assess this construct at 8 and 14 months. Episodic memory can be assessed reliably and efficiently using the NIH Toolbox Picture Sequence Memory Test from age 3 years and continuing into adulthood (28).

### Memory Measurement Plan

Table 2 shows the measurement plan for this construct. We planned to assess recognition memory at 8 and 14 months, and then again at 3, 4, 5, 9, 13, 17, and 21 years. Episodic memory would be measured using an imitation paradigm (in which participants reproduce at demonstrated sequence of actions) at 21 months. The NIH Toolbox Picture Sequence Memory test would be used at 3, 4, 7, 9, 11, and 19 years.

**Table 2:** National Children's Study measurement plan for memory.

**Domain**	**Construct**	**Measure**	**0–1 month**	**8 months**	**14 months**	**21 months**	**3 years**	**4 years**	**5 years**	**7 years**	**9 years**	**11 years**	**13 years**	**15 years**	**17 years**	**19 years**	**21 years**
Cognition	Memory	Recognition memory		X	X												
Cognition	Memory	Episodic memory				X	X	X		X		X		X		X	
Cognition	Memory	Working memory		X	X	X	X	X	X		X		X		X		X

## Language

### Definition

Early language development involves changes in various processes. Early *vocabulary production* (or expressive vocabulary) refers to words that are said and gestures that are formed to communicate. *Vocabulary comprehension* (or receptive vocabulary) refers to words that are understood. Most infants begin to produce some words and gestures late in the first year, and their early production vocabulary is assumed to be a relatively small subset of their comprehension vocabulary [e.g., see (36)]. For example, an infant who produces only 3 or 4 recognizable words or gestures is likely to comprehend the meaning of 30–40 words. *Reading* (including early pre-literacy) refers to a range of skills leading up to and including reading with comprehension.

### Significance

Research indicates that children vary widely in their rate of vocabulary growth (37), and that various aspects of early vocabulary growth have long-term implications for subsequent language development and success in important domains (e.g., classrooms). For example, infants' gesture use at age 14 months predicts vocabulary size at 42 months (38), and early vocabulary size predicts outcomes, such as expressive vocabulary at ages 4 and 5 years (39) and language and literacy achievement up to fifth grade (40). Recent research indicates the importance of assessing the developmental trajectory of vocabulary growth. For example, the rate of vocabulary growth between 14 and 46 months (not just vocabulary size at any one age) predicts later vocabulary at 4.5 years, particularly for children from low-socioeconomic status backgrounds (41). Language comprehension and reading are important components of academic achievement that provide a gateway to learning.

### Feasibility

A widely used technique for assessing a child's vocabulary is to ask parents whether their child produces and/or comprehends particular words or gestures. This technique has been particularly useful for measuring production vocabulary when parents are asked to select from a categorized list of words that infants might say (42, 43). Parent-reported vocabulary has the weakness, however, of being susceptible to subjective bias (e.g., exaggeration by parents who over-interpret the linguistic sophistication of their child's verbal output).

Parental assessment of comprehension is more problematic. Comprehension tends to precede production, and parents are unlikely to gain accurate awareness of words that their children comprehend but do not yet utter. At the same time, parents may systematically over- or underestimate their children's presumed comprehension. Thus, while comprehension can be assessed using parent report, there is some advantage to assessing early comprehension directly. One approach is to show the child an array of pictures, ask the child, "Where is the ___?” and code where the child looks. This looking-while-listening technique has been widely used with infants and toddlers to demonstrate developmental differences in comprehension, preservation of individual differences over time, construct validity when compared with parent report, and good test-retest reliability (44).

Vocabulary comprehension can be assessed reliably and efficiently using the NIH Toolbox Picture Vocabulary Test from 3 years of age and continuing into adulthood, while reading can be assessed effectively beginning at about age 7 years with the NIH Toolbox Oral Reading Recognition Test (45).

### Language Measurement Plan

Table 3 shows the measurement plan for this construct. We planned to measure vocabulary comprehension at 8, 14, and 21 months, and at 3, 4, 5, 9, 13, 17, and 21 years; vocabulary production at 8, 14, and 21 months, and at 3 years; and reading (recognition/decoding) at 4, 7, 11, 15, and 19 years. A measure of reading comprehension is also recommended at age 17 years, however, because reading comprehension is potentially an important outcome variable, related to academic achievement and other functional outcomes.

**Table 3:** NCS measurement plan for language.

**Domain**	**Construct**	**Measure**	**0–1 month**	**8 months**	**14 months**	**21 months**	**3 years**	**4 years**	**5 years**	**7 years**	**9 years**	**11 years**	**13 years**	**15 years**	**17 years**	**19 years**	**21 years**
Cognition	Language	Vocabulary comprehension		X	X	X	X	X	X		X		X		X		X
Cognition	Language	Vocabulary production		X	X	X	X										
Cognition	Language	Reading (recognition/decoding)						X		X		X		X		X	
Cognition	Language	Reading (comprehension)^*^													X		

* *A measure of reading comprehension, as opposed to reading recognition, is recommended for consideration in late adolescence if resources exist for its development. This measure of reading comprehension can be considered an important outcome variable, likely related to academic achievement*.

## Processing Speed

### Definition

*Processing speed* (PS) refers to the rate at which mental operations are performed. Slower PS is reflected in delayed reaction times to various stimulus arrays and longer times to perform mental activities. It is closely associated with attentional functions, and may reflect efficiency in short-term memory scanning and inspection times for perceptual comparisons (e.g., new vs. old, or same vs. different).

PS shows a well-documented curvilinear relation with age across the lifespan. Similar to the pattern seen with other cognitive processes, including executive function skills and measures of “fluid intelligence,” PS shows an increase in efficiency across childhood, peaks in early adulthood, and then on average declines rather sharply through older adulthood. During childhood, PS in different types of tasks improves at a common rate (46–49), supporting the notion of their dependency on more global aspects of brain development. In addition, factor analytic studies provide evidence that various measures of PS are significantly intercorrelated and can be distinguished from other related cognitive constructs, such as response inhibition, working memory, and executive function throughout childhood and adolescence (50, 51).

### Significance

PS is a fundamental aspect of cognition that might serve as a rate-limiting factor for more complex cognitive skills and for their healthy development (52). Improvements in PS throughout childhood are associated with improved performance on a broad range of cognitive tasks, including executive function skills, reading, memory, arithmetic problem solving, way-finding, and reasoning (53, 54). In some cases, PS accounts for all of the variance in age-related cognitive changes (i.e., a significant association between age and some aspect of cognitive function disappears when PS is partialled out). In addition, longitudinal research shows that measures of PS administered during infancy predict cognitive development in the preschool years (55).

Research has also shown that measures of PS are among the most sensitive cognitive indicators of general cerebral dysfunction (56). For instance, slowed PS has been demonstrated in traumatic brain injury, Multiple Sclerosis, Parkinson's disease, symptomatic human immunodeficiency virus, dementia, and schizophrenia (57). Further, slowed PS has been associated with changes in neurotransmitter activity (e.g., reduced cholinergic function, reduced D2 receptor sites for dopamine, and altered glutamate activity), white matter integrity, glucose metabolism, and nerve conduction velocities (e.g., as measured by evoked potentials, event-related potentials, and electroencephalography) (58). In addition, children with central nervous system impairments, including developmental disability (58), closed head injury (59), and phenylketonuria (60), have slower PS relative to their typically developing peers. Long-term survivors of childhood acute lymphoblastic leukemia who have been treated with radiation (resulting in myelin damage) have slower PS than controls (61, 62). There also is evidence from neuroimaging studies that increased white matter volumes (used as an index of myelination) are associated with faster PS (63). Taken together, results indicate that PS is vulnerable to impairment by a broad range of injuries or diseases involving the brain throughout the lifespan.

### Feasibility

Prior research has demonstrated reliable assessment of PS in infants using timed gaze in a continuous familiarization task (64), reaction time in a visual expectation task (65), and a novelty preference score in a visual familiarization paradigm (familiarization time needed before showing a reliable novelty preference) (66). For infants and toddlers, measures of PS can be derived incidentally from performance on tests designed to assess other aspects of cognition, such as executive function, spatial cognition, memory, and social cognition. Looking time responses in the youngest children to various stimuli can be recorded and examined for psychometric suitability as a PS composite. This composite would be conceptually similar to the RT composite created for the NIH Toolbox Cognition Battery (52), and has the added advantage of not requiring time to administer additional, speed-specific items. The latter advantage may be especially important for assessment of the youngest children.

The NIH Toolbox Cognition Battery contains a brief discrimination time (same-different judgments) assessment of PS called the Pattern Comparison Processing Speed Test (52), which has been normed and validated for examinees from ages 7 to 85 years. It has demonstrated strong correlations with results on published tests of PS, and shows adequate test-retest reliability in both children and adults. Moreover, in the large NIH Toolbox norming sample of children aged 3–18 years, the Pattern Comparison Test showed good convergent validity (*r* = 0.65) with a composite reaction time score from two Toolbox tests of executive function (Flanker and DCCS) (52).

### Processing Speed Measurement Plan

Table 4 shows the measurement plan for this construct. We planned to measure looking time at 8, 14, and 21 months, and at 3 years; and discrimination time (Pattern Comparison Processing Speed Test) at 3, 4, 5, 7, 9, 13, 17, and 21 years.

**Table 4:** NCS measurement plan for processing speed.

**Domain**	**Construct**	**Measure**	**0–1 month**	**8 months**	**14 months**	**21 months**	**3 years**	**4 years**	**5 years**	**7 years**	**9 years**	**11 years**	**13 years**	**15 years**	**17 years**	**19 years**	**21 years**
Cognition	Processing speed	Looking time		X	X	X	X										
Cognition	Processing speed	Simple discrimination time					X	X	X	X	X		X		X		X

## Spatial and Numerical Processing

### Definition

Spatial cognition, broadly construed, refers to the encoding, manipulation, and use of environmental information (i.e., objects, small-scale configurations, and large-scale, navigable spaces) [e.g., (67–69)]. In human beings, spatial cognition includes behaviors as commonplace as reading a map and as specialized as computing trajectories for the rover Curiosity on Mars. Given the diversity of behaviors supported by spatial cognition, there is growing consensus that it is best understood, particularly over ontogenetic time, when partitioned into well-defined clusters of processes, including: *geometric sensitivity, location coding*, and *mental rotation*. We thus define spatial cognition according to these clusters and briefly describe developmental and individual differences below with reference to each.

1. **Geometric Sensitivity**. Spatial cognition includes the processing of the geometry of individual objects and the geometry of the layout specified by multiple objects (70, 71). Geometric sensitivity encompasses the representation of Euclidean properties, such as length/distance, angle, and sense/direction. Geometric sensitivity supports behaviors, such as shape discrimination, as well as analyses of complex forms and layouts, including comparisons of the relations within and between layouts.
2. **Location Coding**. Spatial cognition also includes the processing of the location of objects, people, and places (72, 73). Location coding involves specifying the location of one or more targets relative to an egocentric and/or allocentric frame of reference. Egocentric reference is specific to the observers themselves (as when an object is specified as being “in front and to my left”), whereas allocentric reference involves landmarks in the external environment and requires use of geometric properties, such as distance and direction (as when an object is specified as being “5 feet southeast of the tower”). To be useful, locations must be maintained in memory and updated following changes in viewing perspective.
3. **Mental Rotation**. Spatial cognition, particularly when assessed psychometrically, is most often associated with mental rotation; that is, visualizing and manipulating images of objects, people, or scenes in one's mind, which allows for images to be compared across different perspectives (74, 75). These comparisons can encompass a range of movements, from translations along a single coordinate (e.g., *x*- or *y*-axis) to rotations that involve multiple coordinates, including movements in depth.

Given the close conceptual connections between spatial and numerical cognition, as well as accumulating evidence of shared neural mechanisms involving posterior parietal cortex, there is growing consensus among researchers interested in normative and atypical development that these domains are best understood when considered not in isolation, but in relation to each other (76). Measurement skills, and concepts, such as general magnitude representation (77) and the mental number line (78), involve both domains. We thus proposed to assess three aspects of numerical cognition: (1) *number estimation*, (2) *measurement*, and (3) *formal math*.

Numerical cognition ranges from basic abilities, such as estimation (without explicit counting) to more complex skills, such as knowledge of counting routines as well as formal measurement and arithmetic (79). Human and many non-human animals share the capacity to engage in non-verbal number estimation, and this ability is supported by an approximate magnitude system dependent on neural networks involving the intraparietal sulcus, which represents numbers in analog format (80). Although limited in precision, this system has been found to play an important role in the acquisition of symbolic number processes, such as counting and arithmetic (81). The acquisition of measurement skills builds on other basic concepts, such as the mental number line, which changes over the course of childhood (79).

The seeds of spatial and numerical cognition can be measured in infancy. Within the first year, infants discriminate and categorize objects according to shape (82). They also use simple environmental cues, such as the configuration of 2-D forms (83) and distance along a single axis to localize a target (84). Rudimentary mental rotation for 2-D and 3-D objects has been reported in infants between 3 and 9 months (85, 86), though individual differences in performance remain poorly understood (74, 87). In the case of numerical cognition, infants 6–11 months of age not only discriminate arrays of objects that differ in number, but they also represent numerical values according to their ordinal relations (79).

Developmental changes in geometric sensitivity, location coding, mental rotation, and numerical cognition are well-documented within the first 2 years of life, with substantial improvements during the preschool and elementary school years. Studies indicate improvements in location coding, both with respect to the number of locations children can track and the delays they can withstand (88). Children also come to handle more complex allocentric cues, such as distances along multiple, rather than single, axes (89), and they eventually combine egocentric and allocentric reference frames in optimal (Bayesian) fashion (90). Developmental changes in mental rotation include increases in speed and the complexity of rotation (74). In the case of numerical cognition, significant strides are typically made during the preschool and school years in the acquisition of symbolic number concepts, such as the acquisition of number words, Arabic digits, the use of counting for identifying cardinality (i.e., knowing that the word “five” applies to exactly 5 objects, not 4 or 6) and understanding the successor function (i.e., knowing that the number 5 has only one unique successor, namely, the number 6). Children also tend to acquire a vast range of formal arithmetic concepts, with increases in computational speed and shifts from external to internal mental computations (91, 92).

Individual differences in spatial and numerical cognition are well-documented throughout development, beginning in infancy and continuing into adulthood (93, 94), and children from lower socioeconomic status groups perform worse than children from higher socioeconomic status groups on a variety of measures (95, 96). Socioeconomic status differences can be seen as early as the preschool years and remain stable or increase in magnitude by elementary school age (97, 98).

### Significance

Different aspects of spatial cognition, such as geometric sensitivity and mental rotation measured in childhood have been found to be predictive of the likelihood of going on to achieve advanced education credentials and occupations in STEM (Science, Technology, Engineering, and Mathematics) fields, even when controlling for other relevant abilities, such as verbal competence (99). Longitudinal research has found that spatial cognitive abilities measured in elementary school predict creativity and technical innovation later in life, as measured by patents and refereed publications (100). Other studies demonstrate even earlier links between spatial cognition and STEM success (93). Moreover, there is evidence that training mental rotation ability results in improved arithmetic competence in school-age children (101).

Children with delays in learning formal math concepts may be diagnosed with dyscalculia. Children with dyscalculia are generally identified because of difficulties comprehending arithmetic, but their deficits often include more basic abilities, such as non-verbal number estimation (102). In addition to specific learning deficits in math, these children may go on to perform poorly in science and to experience social stigma associated with having a learning disability (103). Recent studies suggest that even basic numerical competence is associated with better occupational prospects and higher income, as well as greater life satisfaction, happiness, and general well-being, even after other cognitive and social factors are controlled (104).

Individuals who struggle with spatial and numerical abilities are at risk for underachieving at school, not securing employment, and lower overall quality of life. These issues not only lead to large personal costs, but also to substantial financial burdens to governments (104). Fortunately, accumulating evidence from training studies suggests that spatial and numerical cognition are not immutable. For example, exposure to puzzles in early childhood and practice with videogames, such as Tetris improve mental rotation performance on standardized tests, and feedback during ordinal tasks (e.g., “Which array contains the larger number of objects?”) improves the precision of these judgments, such that smaller differences in numerosity become discriminable (105, 106). Given the reach and impact of spatial and numerical cognition on both our intellectual and social lives, it is increasingly critical that projects, such as the NCS examine the integrity of these constructs from early in development.

### Feasibility

The six spatial and numerical constructs we identified for assessment beginning in infancy and across development into adulthood can be measured straightforwardly using standard experimental procedures and a variety of dependent measures [e.g., (82, 84, 93)]. In infancy, these spatial and numerical constructs are assessed using animated displays presented on a computer tablet equipped with a camera for recording looking behaviors, such as anticipatory looking or preferential looking (93). To assess geometry sensitivity, for example, an initial set of trials display a shared property (e.g., shape), and then on subsequent trials, we measure infants' looking behaviors to a novel scenario that requires differentiating between categories (e.g., triangle vs. square). In this example, a character moves behind an occluder on each familiarization trial, with each trial representing a distinct exemplar for the shape category (e.g., three different triangles serve as occluders); during test trials, we measure anticipatory looking toward one of two occluders, one from the same shape category (i.e., novel triangle) and a distractor from a different shape category (e.g., square). A measure of location coding similarly assesses anticipatory looking during test trials, following familiarization to an array of objects (e.g., three containers oriented horizontally) that appears in different positions on the tablet, ensuring an allocentric, not egocentric, target location. Measures of mental rotation and non-verbal number estimation based on preferential looking exist for infants and are predictive of spatial and numerical performance on paper-and-pencil tasks at preschool age (93, 107). In infancy, these tasks are modeled on change detection paradigms, in which two dynamic displays (left and right) vary in the amount of variability. In the mental rotation task, one display includes an object presented from different orientations, whereas the other display also presents the mirrored object. Previous research demonstrates that infants look longer toward the display with the mirrored object, consistent with change detection based on mental rotation. In the non-symbolic number task, one display presents an array of objects that differ in their positions and physical sizes, whereas the other display also presents arrays that differ in number (e.g., alternating displays of 10 and 20 dots). Previous research demonstrates that infants look longer toward the displays with alternating numerosities, when the difference is discriminable (107).

Measures for toddlers, preschoolers, older children, and adults include portable and shortened versions of empirical assessments used in the existing literature [e.g., (72, 94, 96–98)]. These assessments, presented on a tablet, range from game-like localization tasks for young children to puzzle-like problem solving tasks yielding considerable data (e.g., reaction time data) regarding spatial and numerical processing.

### Spatial and Numerical Processing Measurement Plan

Table 5 shows the measurement plan for this construct. We planned to measure geometric sensitivity at 8 and 21 months, and at 3, 5, and 9 years; location coding at 8 and 21 months, and at 3, 4, and 7 years; number estimation at 8 and 14 months, and at 3, 5, and 9 years; mental rotation at 8 and 14 months, and at 3, 5, 9,13, 17, and 21 years; measurement at 3, 4, and 5 years; and formal math at 7, 11, 15, and 19 years.

**Table 5:** NCS measurement plan spatial and numerical processing.

**Domain**	**Construct**	**Measure**	**0–1 month**	**8 months**	**14 months**	**21 months**	**3 years**	**4 years**	**5 years**	**7 years**	**9 years**	**11 years**	**13 years**	**15 years**	**17 years**	**19 years**	**21 years**
Cognition	Spatial and numerical	Geometric sensitivity		X		X	X		X		X						
Cognition	Spatial and numerical	Location coding		X		X	X	X		X							
Cognition	Spatial and numerical	Number estimation		X	X		X		X		X						
Cognition	Spatial and numerical	Mental rotation			X	X	X		X		X		X		X		X
Cognition	Spatial and numerical	Measurement					X	X	X								
Cognition	Spatial and numerical	Formal math								X		X		X		X	

## Social Cognition

### Definition

Social cognition (i.e., understanding self and other persons) is needed to navigate our complex, human social world. The development of social cognition begins early, as reflected in the *perception and recognition of persons* (e.g., faces, emotions), *social responsiveness* (e.g., joint attention; someone looks at an object and the infant follows that gaze to look at the same object), and *understanding of intentional actions* (i.e., of goal-directed behaviors). Much social cognition centers around a construal of self and others in terms of mental states (e.g., beliefs, desires, intentions, emotions), called *theory of mind;* in colloquial terms, this involves “putting oneself in other peoples' shoes” (appreciating how the other person is likely feeling, interpreting, or reacting to a situation). As adults, we typically construe people as engaging in actions they *believe* will get them what they *desire*: Jill watched TV because she *wanted* entertainment and *thought* a good program was on. Theory-of-mind understandings begin in infancy, and they develop across childhood, as children come to understand more about internal mental states, especially beliefs and desires. A classic assessment for this uses false-belief (FB) tasks. For example: Max places chocolate in the drawer, and when he goes away it is moved to the cupboard. When he returns, where will he look? Success on such tasks shows understanding of crucial distinctions between what's mental and what's real (Max will search in the drawer but the chocolate is really in the cupboard), and a grasp of mental-state subjectivity (I think the chocolate is in the cupboard but Max thinks it's in the drawer). Meta-analyses of hundreds of studies show very young preschoolers fail false-belief tasks (yet succeed on warm-up, comprehension, and control questions) whereas 4-, 5-, and 6-year-olds pass them, and this is true (with modest variations in timing) in communities worldwide (108).

#### Developmental Sequences

Although they fail false-belief tasks, younger children understand crucial aspects of desires and preferences. This is apparent not only in laboratory tasks (109), but also in children's acquisition and use of *mental state terms* for talking about people. Two- and 3-year-old children talk often about persons' desires via terms like *want, need, like*, but rarely talk about persons' beliefs. By age 4 and 5 years, children also use terms like *think, know, remember*, and *guess*. A pattern of earlier understanding of desires and later understanding of beliefs is apparent in the conversations of US children, Chinese children (110), Spanish-speaking children (111), and even deaf children speaking sign language (112).

Understanding desires and false beliefs are just two milestones in the protracted development of an extended theory of mind. A standardized Theory of Mind Scale assesses childhood understanding of (1) diverse desires, or “DD” (people can have different desires for the same thing), (2) diverse beliefs, or “DB” (people can have different beliefs about the same situation), (3) knowledge-access, or “KA” (something can be true, but someone might not have access to that information and so be ignorant), (4) false belief, or “FB” (something can be true, but someone might believe something different), and (5) hidden emotion, or “HE” (someone can feel one way but display a different emotion). US preschoolers evidence a clear order of difficulty [as listed above DD>DB>KA>FB>HE; (113)] as do Australian, German, French, Turkish, and Indonesian preschoolers. A very similar (but culturally shaped) sequence characterizes children in China (113) and Iran (114). Deaf children of hearing parents exhibit the same sequence, but greatly delayed, and children with autism are equally dramatically delayed, but moreover exhibit a different sequence (115). This Theory of Mind Scale has been extended to later steps focused on children's understanding of non-literal language (NL), such as sarcastic jokes and shows progressive developments in typically developing children up until about 12 or 13 years, and in delayed individuals (e.g., those with deafness or autism) into adulthood (116).

In summary, we identified a total of four proposed social cognition constructs:

1. *perception and recognition of persons* (e.g., faces)
2. *social responsiveness* (e.g., orienting to one's own name, orienting to another's point)
3. *mental state terms* (including non-literal language)
4. *theory of mind* (understanding behavior in terms of mental states)

### Significance

Social cognition is crucial for the achievement of abilities to communicate, to make friends and affiliations, to cooperate and deceive, to learn from and teach others. Differences in theory of mind understanding, for example, longitudinally predict differences in children's play with their peers, their popularity and social interactions with others including their lying and secret-keeping, and parents' and teachers' ratings of social competence (117–119). Infant social-cognitive understandings longitudinally predict later preschool false-belief understandings even after IQ, language competence, and executive function skills are controlled [e.g., (120)]. Preschool theory of mind predicts children's transition to peer-groups when they later enter school (121), and theory of mind differences correlate with social isolation and difficulties in peer relations in adolescence, for typically developing children and delayed individuals, such as those with autism or deafness (122). Impairments in social cognition are associated with numerous disorders, including autism (123) and antisocial personality disorder (124).

### Feasibility

Standard infant tests exist for the infant constructs, and many of these involve simplified displays (presented on a tablet computer) or parent report measures. For example, eye tracking can be used to assess infants' perception and recognition of persons (125), and for social responsiveness, parents can be asked if the infant “orients/looks when his/her name is called” (this can be tested quickly at an assessment visit as well). Mental state language (for toddlers, preschoolers, and older children) can be assessed via the standardized MacArthur Bates Communicative Development Inventory [CDI; e.g., (37)], a parent-report measure, and tallied from a recording of speech during the assessment visit. The Theory of Mind Scale provides a validated short measure of 6 progressive theory of mind constructs (e.g., DD, DB, KA, FB…) encompassing understandings from 30 months to adolescence, and can be administered via a computer tablet displaying vignettes and via computer adaptive testing (CAT) methods where an abbreviated subset of tasks are presented until consistent successes and failures are found [(116); see section Project 2: Design and Development of a Computerized Version of the Theory of Mind Scale].

### Social Cognition Measurement Plan

Table 6 shows the measurement plan for this construct. We planned to measure perception and recognition of persons at 8 and 14 months; social responsiveness at 8, 14, and 21 months; Theory of Mind Scale Item DD at 21 months; Theory of Mind Scale Items DD, DB, KA FB at 3 years; Theory of Mind Scale Items KA, FB, HE at 4 years; Theory of Mind Scale Items FB, HE, NL at 5 years; understanding of mental state terms at 21 months, and 3, 4, 7, 11, 15, and 19 years; and social decision making at 3, 5, 9, 13, 17, and 21 years.

**Table 6:** NCS measurement plan for social cognition.

**Domain**	**Construct**	**Measure**	**0–1 month**	**8 months**	**14 months**	**21 months**	**3 years**	**4 years**	**5 years**	**7 years**	**9 years**	**11 years**	**13 years**	**15 years**	**17 years**	**19 years**	**21 years**
Cognition	Social	Perception and recognition of persons		X	X												
Cognition	Social	Social responsiveness		X	X	X											
Cognition	Social	Theory of mind scale item DD				X											
Cognition	Social	Theory of mind scale items DD, DB, KA FB					X										
Cognition	Social	Theory of mind scale items KA, FB, HE						X									
Cognition	Social	ToM scale items FB, HE, sarcasm							X								
Cognition	Social	Mental state terms				X	X	X		X		X		X		X	
Cognition	Social	Social decision making					X		X		X		X		X		X

## Next Steps

The measurement plans described above were made by a team of domain experts, and they were designed to provide high-priority information about the nature of neurocognitive development from birth to adulthood as part of a large-scale longitudinal study of outcomes of genetic and environmental influences on cognition. These plans can inform the design of other longitudinal studies that include assessment of cognitive function from birth to adulthood.

The NIH Toolbox Cognition Battery works well for many typically developing children starting around ages 3 or 4 years and up, but is not designed to assess all relevant aspects of cognition, such as social cognition, and numerical and spatial processing. Thus, there was a recognized need for new well-validated and standardized direct behavioral measures of cognition that can be used in large-scale longitudinal studies, especially from birth to age 3 years. Three additional and ongoing projects were focused on the development of new behavioral measures for use in such studies, and we encourage the further use, validation, and refinement of these new measures. Deliverables from these projects (e.g., more detailed plans and examples) are in the public domain and can be found at: https://www.nichd.nih.gov/research/supported/NCS.

### Project 1: Developmental Cognitive Profiler (DCP): Design and Creation of a Computer Tablet-Based Assessment of Cognitive Constructs in Infants and Toddlers

**Figure 1:**
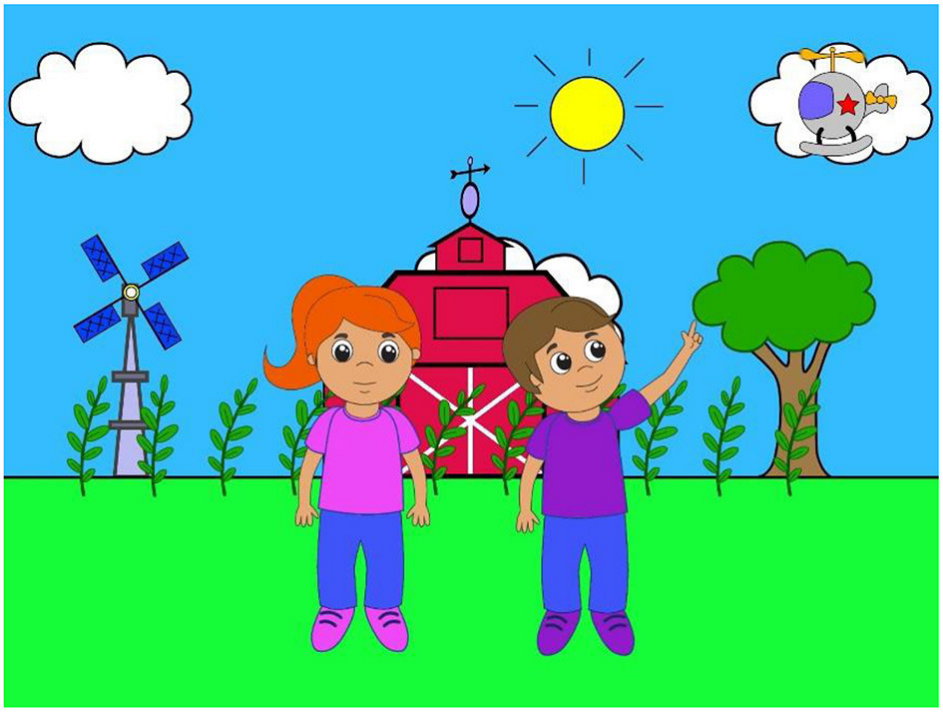
A screen shot from an animated sequence designed to measure an aspect of social cognition (social sensitivity) in infants and toddlers.

**Figure 2:**
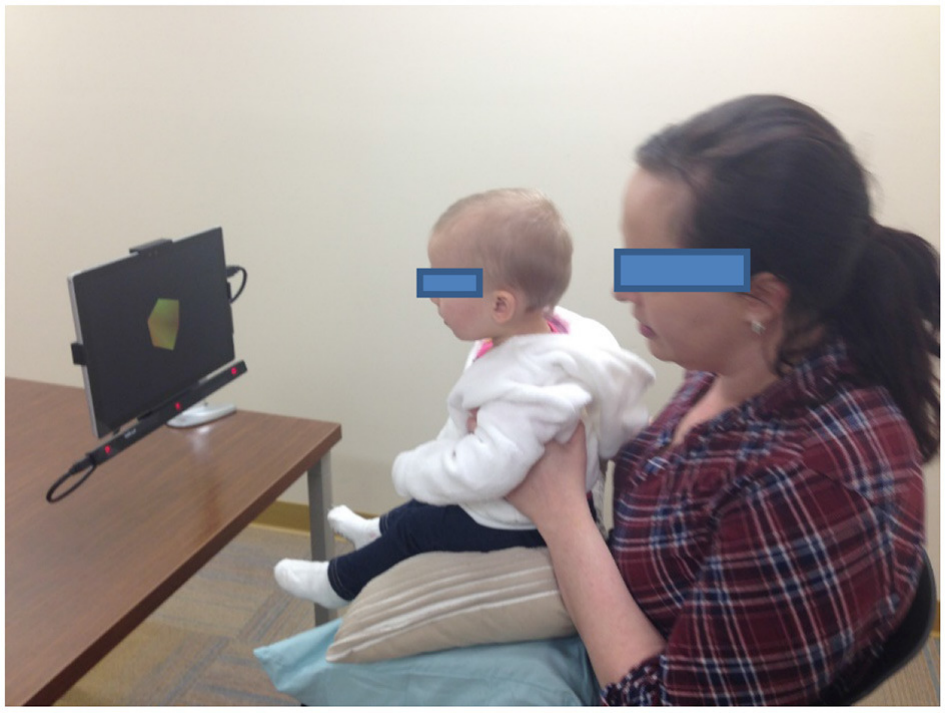
A prototype of the Developmental Cognitive Profiler, designed to assess cognition in infants and toddlers by recording gaze in response to animated sequences. Infants are seated on their parent's lap ~60 cm from the tablet. Parents wear infrared glasses.

This Cognitive Health Domain Team project was designed to create standardized assessments of cognition (i.e., executive function, vocabulary, spatial and numerical processing, processing speed, and social cognition) that can be administered via a portable software platform in 15 min or less to infants and toddlers. Animated event sequences (trial sequences) were designed to elicit specific looking and/or reaching patterns indicative of the underlying cognitive constructs. A set of animated event sequences was developed (see Figure 1) for use on a computer tablet (see Figure 2). The current version of DCP has been refined through an iterative process of testing and tweaking, and is now in validation. Additional potential refinements include:

- Establish norms for the measures (in English)
- Develop manuals and training materials
- Develop multiple forms of the measures
- Develop Spanish and other language versions and norms
- Refine the automated scoring algorithms
- Develop a computer-adaptive version of the DCP measures

### Project 2: Design and Development of a Computerized Version of the Theory of Mind Scale

This Cognitive Health Domain Team project involved the creation of a computerized (tablet-based) version of the Theory of Mind Scale (110), a well-validated tabletop test for preschool age children, along with a manual and training materials. The original scope of the project allowed for development of a 6-level version and the validation of the tablet-based measure but was subsequently revised to develop a functional 5-level version that can now be used in try-out research, revised, and subjected to validation and norming.

In the existing Theory of Mind Scale (109, 116), children are presented with short (~2 min) vignettes (acted out using small props) and typically progress through 6 steps in a standard order (i.e., DD>DB>KA>FB>HE>NL). When cross-sectional samples of children are tested, that order reflects the order of difficulty (with many children, including younger ones, passing #1, fewer and only older ones passing #s 4 and 5). In longitudinal testing, individual children typically proceed up the scale in order.

For the NCS, a computerized 5-level version of the standardized Theory of Mind Scale was developed. This scale assesses childhood understanding of (1) diverse desires (DD), (2) diverse beliefs (DB), (3) knowledge-access (KA), (4) false belief (FB), and (5) hidden emotion (HE), which show a clear order of difficulty (as listed) in US, Australian, German, French, Turkish, and Indonesian preschoolers. There are 2 versions of each of the five scenarios, one in a man's voice and one in a woman's voice. These were tested on a tablet computer with 9 children ranging in age from 4 to 9 years. These versions yielded the standard scale pattern described above for the 5-step Scale (i.e., order of difficulty DD>DB>KA>FB>HE). The team also developed a user's manual to direct assessors how to use this version of the Scale, and how to score it (for further information, see https://www.nichd.nih.gov/research/supported/NCS).

These steps alone yield a measure suitable for further validation, and for preliminary use in larger-scale direct behavioral assessments of children's social cognition. Just as for the original scale, children can receive scale scores from 0 to 5 (summing the total correct items for the five items) that are usable in correlational/regression analyses, as well as additional scores useful for more in-depth social cognitive assessments and research (such as research with deaf children or children with autism spectrum disorder, including intervention research where pre-and post-measures include ToM Scale scores).

In a subsequent validation study with a larger sample of children (e.g., *N* = 80–100), it is recommended that at least a sub-sample of children would receive both the animated tablet version and the original props-and-figurines version of the ToM Scale to directly assess comparability; and a further sub-sample of children would receive alternative versions of the animated version (those with a different mixture of the male and female voices) to establish initial test-retest reliability.

In addition, developing new animations with professional voice recordings for vignette 6 (NL) would allow for broader assessment of ages 11–15 years with this measure. A further useful next step would be adaptation for individualized computer administration (CAT, computer-adaptive testing), so that early responses determine the exact scenarios that are presented (a subset of the 6 possible). Finally, an additional future step should be adapting the animated version for possible large-scale on-line administration.

### Project 3: Executive Function Formative Project: Design and Creation of a Developmental Extension (DEXT) of the Executive Function (EF) Measures of the NIH Toolbox Cognition Battery

This project was designed to develop robust and brief measures of executive function (EF) for the NCS that are suitable for a more diverse range of children at the lower end of the ability range, and as young as a mental age of 36 months. This also involved revising the training materials for these measures. This project has yielded two computer tablet-based tasks, the Flanker—Developmental extension (Flanker-DEXT) and the Dimensional Change Card Sort—Developmental extension (DCCS-DEXT), as well as a revised version of the Children's Behavior Questionnaire Very Short Form (CBQ-VSF) (126), which includes a new supplemental scale to serve as a parent report EF measure (CBQ-VSF+EF). The two tablet-based tasks were designed for touchscreen administration as developmental extensions of the comparable NIH Toolbox tasks (for a more diverse sample of children, including those having difficulty with the regular Toolbox versions of Flanker and DCCS). These new measures of EF are fully integrated with the existing Toolbox measures, such that children who fail minimal criteria on the standard Toolbox EF measures are administered the easier DEXT portion of the instrument. Flanker-DEXT contains 30 items and DCCS-DEXT contains 40 items.

The original goal of the DEXT study was to develop, refine, and validate the EF measures, develop materials required to train test administrators, and publish the results of this work. The scope of the project was revised in 2015 such that the measures would be completed and programmed for administration on an iPad, along with manuals and materials, but no validity or reliability data would be collected.

The developmental extensions of both NIH Toolbox EF measures have been constructed for use on an iPad, and they are accessible through the Apple App Store. These measures are available for use in studies that assess their validity and reliability for a wider range of ages and socio-economic backgrounds (29, 127). Tentative scoring (a sum total of DEXT items answered correctly) has been developed, but will likely be adjusted following the first wave of validation studies.

We recommend a full-scale validation study of the new measures with respect to time burden, usability, reliability, and construct validity in a nationally representative sample of diverse families with preschool-aged children ages 2.5–5.5 years of age. Data analyses should examine correlations of new measures with age, each other, other measures of EF, traditional IQ measures, and criterion measures of school readiness (literacy and numeracy) that have established predictive validity, both overall and with age covaried. Analyses should also examine relations of child scores to parent education level, wealth, income, and other demographic variables.

These validation data would inform future refinements of the measures and scoring protocols, and would provide a foundation for further work to validate the measures in other languages or populations of interest, and to validate them for additional purposes, including early childhood screening and diagnostic purposes. A subsequent step is to create national norms for the new measures using a stratified representative sample.

## Discussion and Conclusion

The healthy development of cognitive function, coincident with healthy brain development, is essential for successful adaptation. As such, it is a key dimension of human development to consider both as an outcome of environmental and other influences, and also as a potential protective factor that supports resilience despite risks for adverse outcomes. The National Children's Study Cognitive Health Domain Team designed a plan for assessing the development of cognition longitudinally from infancy through adulthood using a paradigm-based approach in infancy and toddlerhood, and relying largely, but not exclusively, on the NIH Toolbox Cognition Battery starting at around 3–5 years. This plan included direct behavioral assessments of (1) executive function skills, (2) memory, (3) language, (4) processing speed, (5) spatial and numerical processing, and (6) social cognition, administered longitudinally from early infancy into adulthood.

Based on this plan, three research and design projects focused on the development of new direct behavioral measures of cognition that can be used in large-scale longitudinal studies, especially from birth to age 3 years. The projects included: the design and creation of computer-tablet-based and paradigm-based assessment of cognitive constructs in infants and toddlers (the DCP); the design and development of a computerized tablet version of the Theory of Mind Scale; and the design and creation of a Developmental Extension (DEXT) of the executive function measures of the NIH Toolbox Cognition Battery, together with the creation of a revised version of the Children's Behavior Questionnaire Very Short Form (127), which includes a new scale measuring parent-reported executive function skills. More information about these measures is available at https://www.nichd.nih.gov/research/supported/NCS.

An important feature of the plan for assessing cognition as part of the NCS is the efficiency and practicality with which a wide range of variables can be measured using standardized procedures in which data are recorded automatically. The paradigm-based approach in infancy and toddlerhood relies on well-established laboratory measures of cognition (e.g., visual expectation, delayed response, looking while listening) that have been adapted for use on a computer tablet. With carefully selected trial sequences, several different skills can sometimes be assessed simultaneously, and eye tracking data can be scored using automated scoring algorithms. Starting in early childhood, these same skills can then be measured efficiently and reliably using the NIH Toolbox Cognition Battery, as well as the newly developed computerized version of the Theory of Mind Scale. We believe that these plans can inform the design of future longitudinal studies of cognitive development, and we encourage the use of comparable designs and measures across studies, which has the potential to contribute to a more globally representative characterization of how the development of cognition unfolds.

Other standardized assessments of cognition in infancy and toddlerhood, such as the Bayley Scales of Toddler and Infant Development (128) and the Mullen Scales of Early Learning (129), require high levels of training on the part of the examiners, depend on children's motor skills, and generally take far longer than 15 min. These measures yield relatively global assessments (e.g., cognition, language, motor), may not capture more subtle individual differences, and often fail to predict longer-term outcomes [e.g., (130)].

## Data Availability Statement

Publicly available datasets were analyzed in this study. These data and other deliverables generated for this study can be found at: https://www.nichd.nih.gov/research/supported/NCS.

## Author Contributions

PZ, SL, MF, JE, RH, HW, JS, MK, and JR drafted the sections of the article and edited the revisions. All authors worked collaboratively as part of the National Children's Study Cognitive Health Domain Team.

## Conflict of Interest

The authors declare that the research was conducted in the absence of any commercial or financial relationships that could be construed as a potential conflict of interest.
